# A Low-Cost Device for Teaching Surgical Techniques and Evaluating Technology in Minimally Invasive Surgery

**DOI:** 10.7759/cureus.116371

**Published:** 2026-09-16

**Authors:** Thomas Hanks, Dominic Nistal, Adam Kosky, David Desmaris, Marc R Mayberg

**Affiliations:** 1 Medicine, University of Washington School of Medicine, Seattle, USA; 2 Neurological Surgery, University of Washington School of Medicine, Seattle, USA; 3 Engineering, Product Creation Studio, Seattle, USA

**Keywords:** cerebrospinal fluid leak, dural repair, endoscopic neurosurgery, minimally invasive surgery, neurosurgery

## Abstract

Minimally invasive surgery (MIS) spans multiple surgical disciplines and offers multiple benefits. Widespread adoption of MIS techniques has been hindered in part by technical challenges from steep learning curves and limited options for low-cost training for surgeons. This technical report describes the design and utility of a 3-D printed MIS simulator as a platform for acquiring skills in MIS techniques, as illustrated by the demonstration and comparison of multiple endoscopic dural repair techniques. The MIS simulator was constructed from 3-D printed and commercially available components, which provided the following attributes: (1) video perspective of the surgical target equivalent to endoscopic visualization; (2) variable access parameters, including portal size and angle of approach; and (3) adaptable surgical target to simulate the anatomy and physical characteristics of a particular surgical scenario. The estimated cost for the simulator was in the range of $200 to $300. The simulator was used to acquire technical skills and compare alternative techniques for MIS endoscopic durotomy repair. It allowed a comparison of different MIS techniques and demonstrated operator competency. The MIS simulator provides a portable, low-cost model for teaching MIS techniques by emulating the technical, anatomical, and physical characteristics of a common MIS encounter. The simulator can be readily modified to include multiple MIS settings and can be used for both training and technical development of instrumentation or novel surgical approaches. Due to its low cost and portability, the simulator is well-suited for care environments where limited tools and materials are available.

## Introduction

The advent of minimally invasive surgery (MIS) in many surgical disciplines has produced significant clinical and economic advantages but has also engendered a host of new technical challenges [1]. Tubular portals and endoscopic approaches in MIS have been associated with reduced length of hospitalization, improved post-operative pain and patient satisfaction, and expanded patient selection [2]. While many patients greatly benefit from these techniques, the restricted operative windows in endoscopic surgery and the need for technical mastery of the instrumentation used in these procedures have hindered widespread adoption of certain MIS approaches [3]. To address these issues, we have developed a simulator that enables a lower-cost, easily reproducible method to train surgeons in the technical skills required for MIS, including the management of common intraoperative complications. Additionally, intraoperative complications associated with MIS techniques, such as inadvertent cerebrospinal fluid (CSF) leaks, are associated with limited options for repair with variable effectiveness [4]. The learning curve for such complications is steep, since the repair must be immediately accomplished during the procedure, and many surgeons have limited experience with MIS and endoscopic techniques. Therefore, an MIS simulator for the repair of CSF leaks may enable trainees or practicing surgeons with limited MIS experience to develop the technical skills necessary for watertight dural closure. 

Traditional methods of surgical simulation for acquiring surgical skills include live animal surgery or human or animal cadaveric specimens, which are often limited by cost and scarcity of resources and host facilities, as well as ethical considerations [5]. This is particularly true in environments with basic tools and limited resources. Low-cost 3-D printed surgical models are usually restricted to addressing a specific pathology, and the soft tissue characteristics do not correspond to the surgical environment, with the loss of important haptic feedback to the surgeon in the learning process [6]. In addition, most of these models lack essential components of MIS, such as the restricted scope of vision and angle of approach through tubular or endoscopic access portals, as well as managing unique features of the surgical field such as bleeding, tissue friability, and anatomical barriers [7,8]. Depending on the procedure, CSF leaks occur frequently during both spine (1%-17%) and cranial (1%-20%) surgeries, and are associated with substantial patient morbidity, as well as increased costs of hospitalization [9]. Current methods for repairing dural perforations in MIS and open procedures broadly include (a) direct suturing or stapling, (b) tissue or synthetic grafts, and (c) tissue sealants, used alone or in combination [10-12]. These techniques are limited in MIS procedures by difficulty of implementation, limited graft visualization and manipulation after deployment, and unreliable dural sealing efficacy [12]. We have addressed these limitations with the MIS simulator described herein, which can easily accommodate different surgical targets and their surrounding anatomy, with the inclusion of important haptic characteristics such as pressurized or non-planar dura.

To improve the quality of training for MIS procedures, we developed an apparatus to provide a low-cost, portable, multimodal device for the teaching of a variety of MIS approaches, showed its effectiveness for the training of consistent and effective MIS dural repair techniques, and highlighted performance trends by the user over time.

This article was previously presented as a poster at the 2025 Congress of Neurological Surgeons (CNS) Annual Scientific Meeting at Los Angeles on October 14, 2025.

## Technical report

Concept and design of the MIS simulator

In this innovation study, we introduce the basic design of the simulator which consists of a lightweight, portable 3-D printed box approximately 10 inches in each dimension that includes (1) an inexpensive, off the shelf 5.5 mm fiber-optic endoscope camera and light source with a video screen analogous to a typical endoscopic view (HD Snake Borescope Inspection Camera; Teslong; Irvine, CA) (Figure 1A);

**Figure 1:**
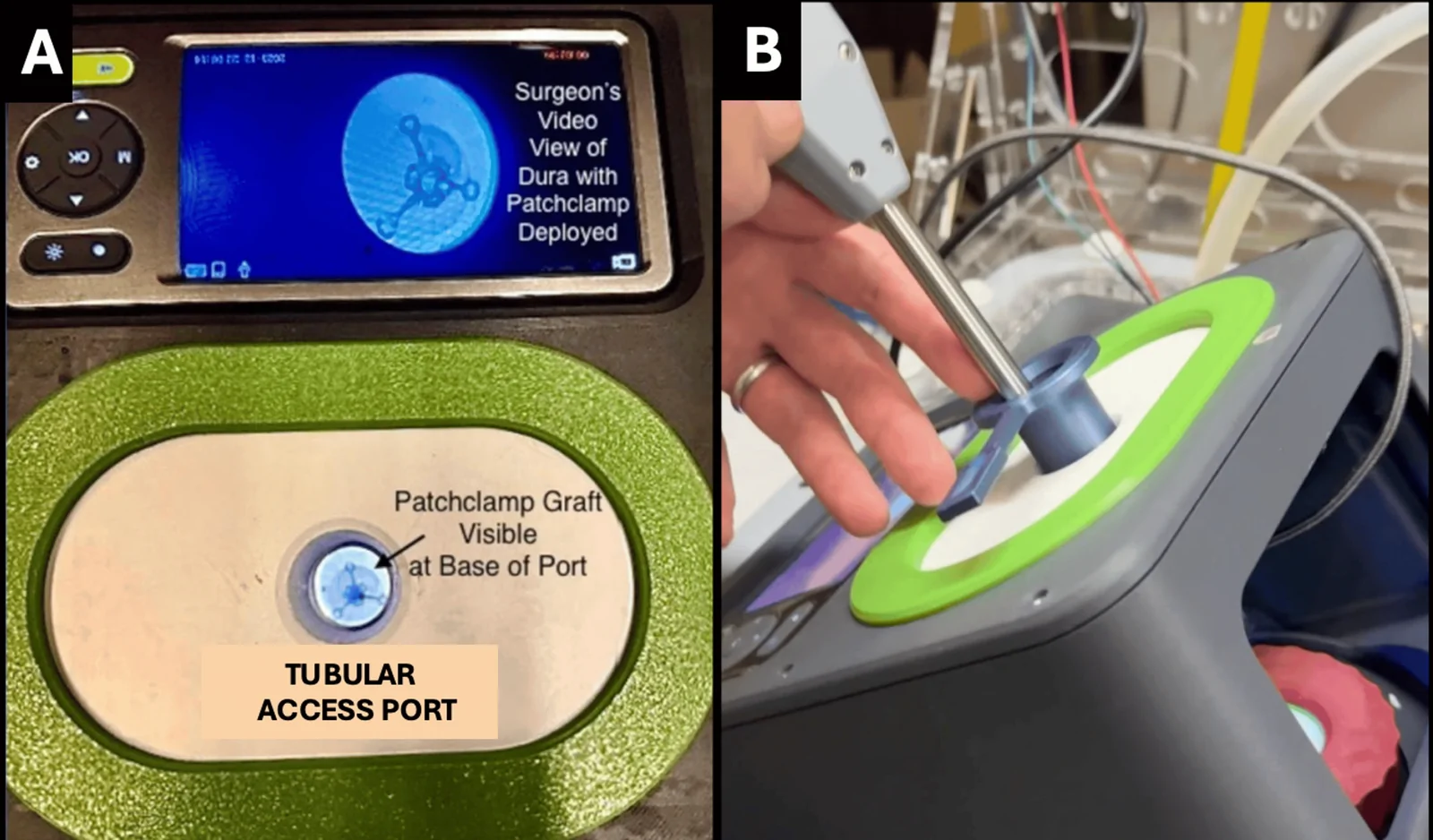
Minimally invasive surgery (MIS) simulator (A) Photograph of the upper surface of the MIS simulator after the implantation of the Patchclamp graft; endoscopic video screen (top) showing the sealing of durotomy by the implant, and the MIS tubular access port (bottom) with the graft visible in the dural membrane; (B) MIS simulator with the Patchclamp graft applicator being placed through MIS port to access the dural membrane in the pressure chamber (red), oriented at a 30° angle.

(2) an access portal to accept cylindrical working channels of 7 mm-24 mm (Figure 2A); and

**Figure 2:**
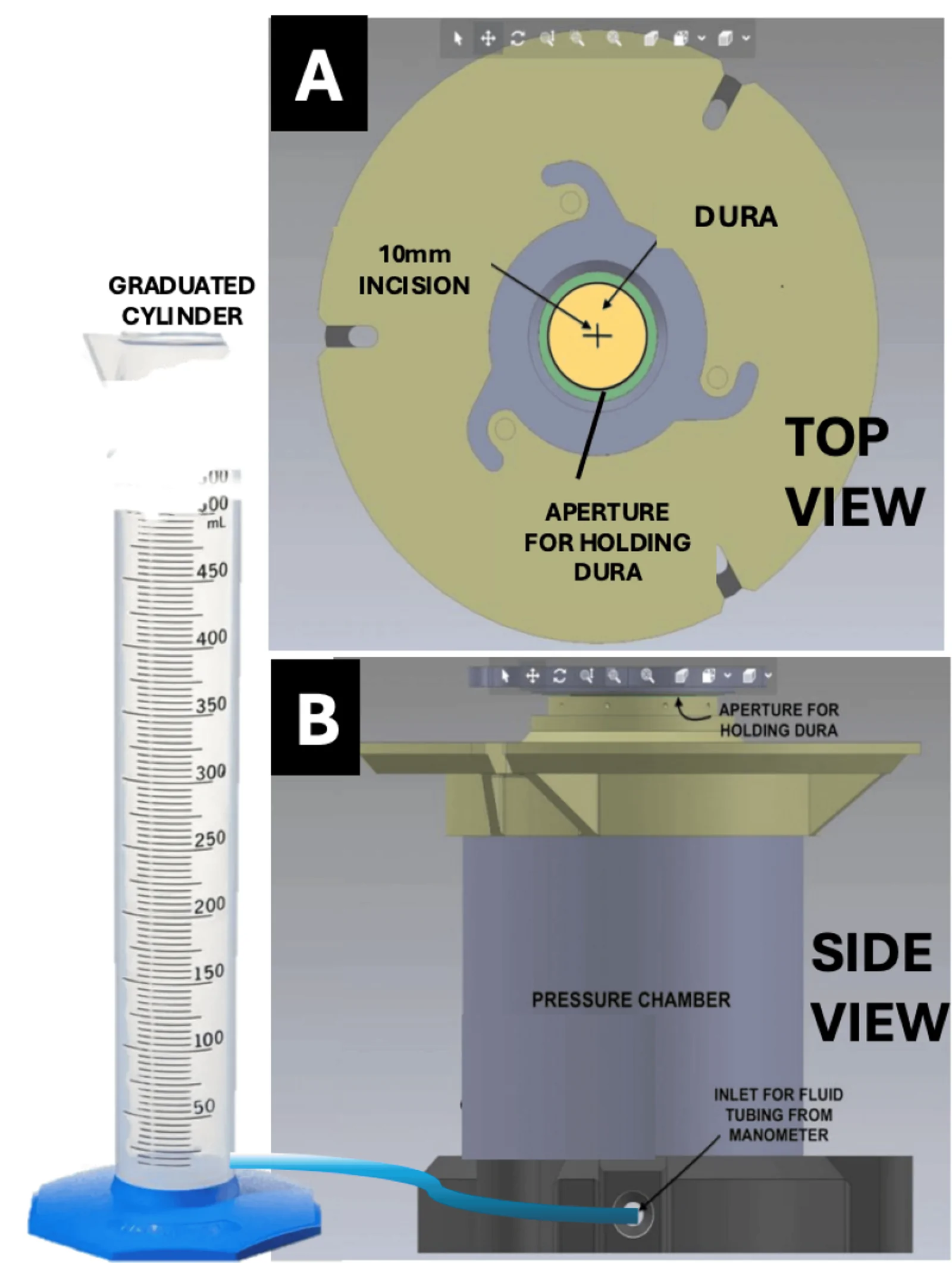
CAD drawings of pressure chamber for the minimally invasive surgery (MIS) simulator (A) Top view showing the dura secured in the aperture and the cruciate durotomy incision; (B) Side view showing the mechanism for pressure regulation by the water column in the graduated cylinder.

(3) a scaffold that accommodates a surgical target specific to the MIS procedure being performed at variable approach angles from perpendicular to a 30° offset (Figures 3A, 3B).

**Figure 3:**
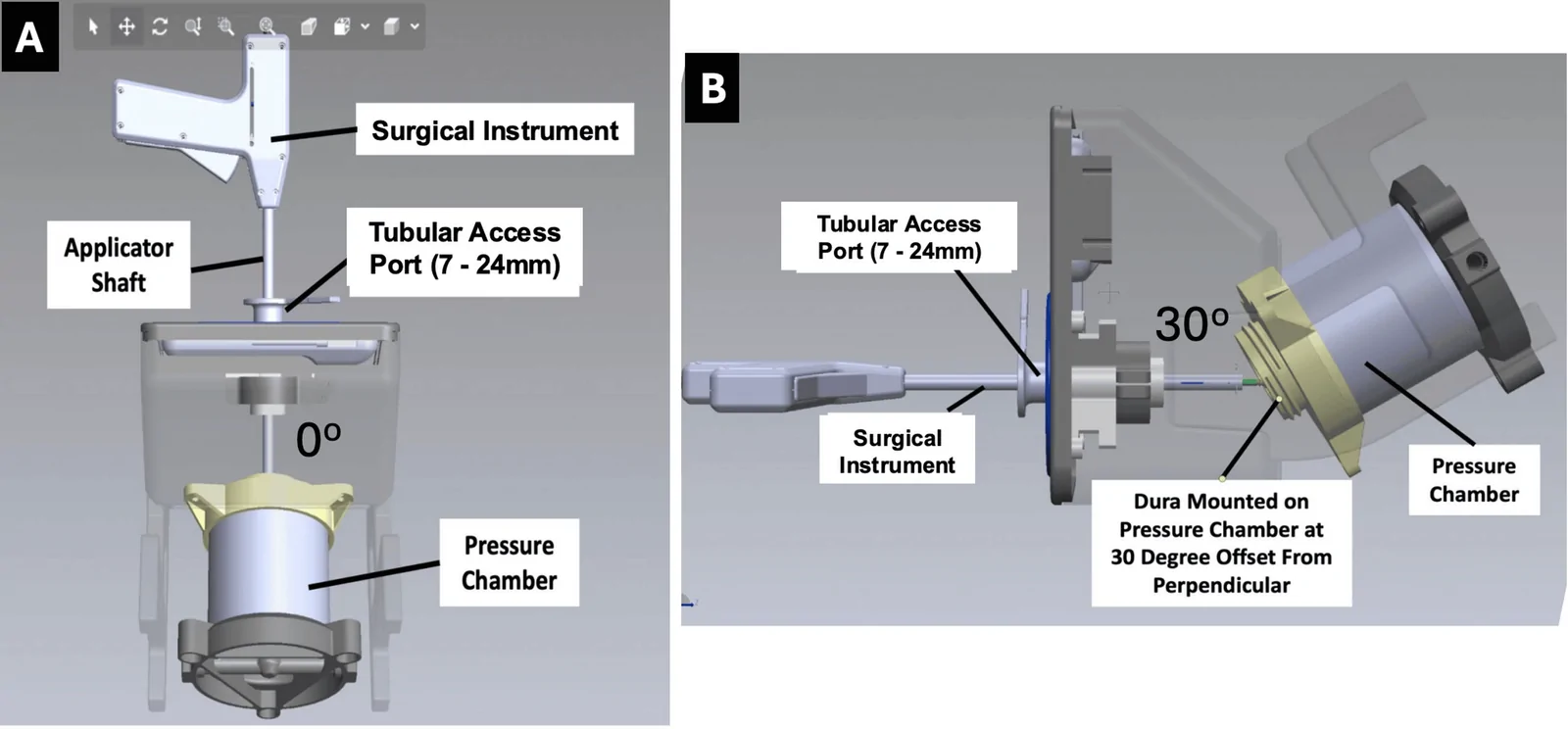
CAD representations of pressure chamber situated in the minimally invasive surgery (MIS) simulator Image shows the approach of the surgical instrument (Patchclamp applicator) via tubular access port to the dural membrane secured in the pressure chamber, situated 6 cm below the simulator frame surface. (A) Dural membrane oriented perpendicular (90°) to the angle of approach, and (B) at a 30° angle of approach.

The surgical target can be anatomical (e.g., cadaver tissue or 3-D printed model), or physiologic (e.g., pressurized fluid to simulate blood or CSF, or electrophysiologic to simulate nervous tissue). In the current study, to simulate MIS repair of durotomy, access portals of 14 mm and 22 mm were placed through an opening in the simulator to a position just above an interchangeable membrane of bovine pericardium (Dura-Guard, Baxter/Synovis, St. Paul, MN). 

To simulate CSF dynamics, the membrane was mounted on an aperture in a 3-D printed pressure chamber (Figure 2A), and a simple fluid pressure system allowed accurate internal pressures at normal and supranormal human CSF pressures. Typical lumbar CSF pressure in the lateral decubitus position ranges between 10-18 cm H₂O, with momentary spikes to 30-40 cm H_2_O during coughing, sneezing, or Valsalva maneuvers [13]. The experimental protocol examined chamber pressures in both the normal (5-18 cm H_2_O) and supranormal (up to 100cm H_2_O) human CSF pressure ranges. The access ports could accommodate surgical instruments such as MIS scalpels, needle drivers, bayonet forceps, MIS drills, and a novel MIS dural repair applicator as discussed later in this manuscript (Figure 3A). In an additional simulation to recapitulate MIS repair of durotomies in spinal surgery, a 3-D printed human L4 spine laminotomy model with an interchangeable pressurized dural membrane cassette in the spinal canal (Figures 4A, 4B) was placed with the curved dural surface below the laminotomy and connected to the pressure regulator.

**Figure 4:**
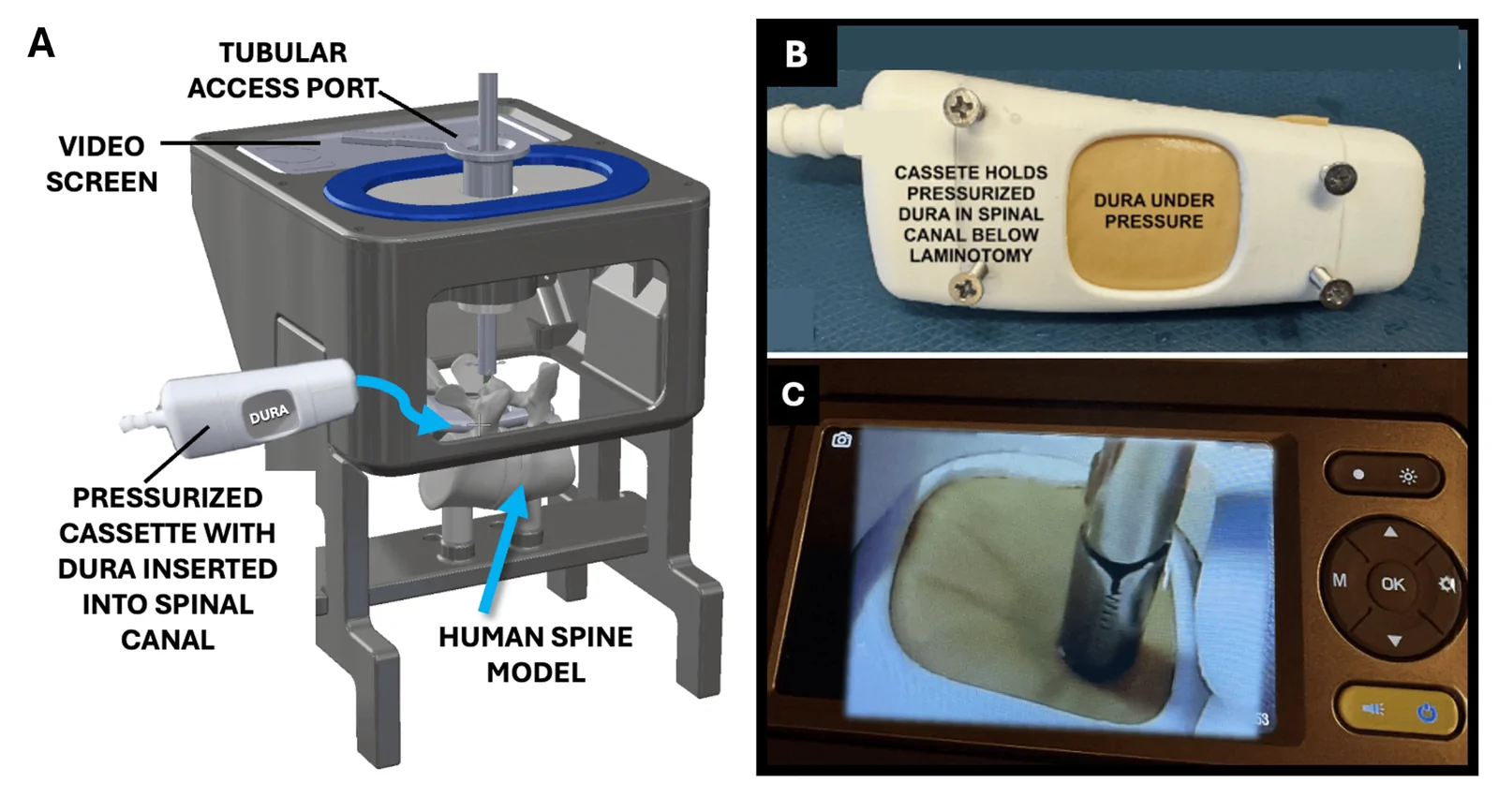
Minimally invasive surgery (MIS) endoscopic spine surgery simulator (A) 3-D printed human lumbar laminotomy spine model placed in the simulator frame below the tubular access port; (B) The cassette is pressurized  from a graduated cylinder via the inlet port and placed in the spinal canal with the curved dural membrane underlying the L4 laminotomy; (C) Video endoscopic view of the surgical instrument indenting the dural surface.

3-D printer, materials, and costs of MIS box production

The MIS simulator enclosure was designed around an inexpensive, readily available boroscope camera system with integrated Light Emitting Diode (LED) lighting and Liquid Crystal Display (LCD) screen, then 3-D printed on a Bambu Labs X1C printer (Bambu Labs, Austin, TX) and used a single spool of carbon fill poly lactic acid (PLA) filament, which costs roughly $40.00. The inexpensive Complementary Metal-Oxide-Semiconductor (CMOS) camera with integrated screen and lighting system was purchased for less than $100.00 and was placed within the MIS simulator to simulate the endoscopic views in MIS surgery. The total costs of production of a single MIS simulator including materials and camera are less than $200.00. Production of the spine model (derived from existing CT spine images) and burst test chamber were also 3-D printed on the same printer, and their costs are limited to the amount of the spool of PLA that was required to print them (approximately $5.00).

Demonstrating technical proficiency for a novel dural repair technique

The MIS simulator provides a realistic platform for the reproduction of intradural surgical procedures, including the repair of incidental and/or intentional durotomies. A waterproof membrane made from bovine pericardium (Dura-Guard, Baxter/Synovis, St. Paul, MN) was selected as the best replica of the human dura. Bovine pericardium has structural characteristics nearly identical to adult human dura and has been used in prior models for simulated dural repair [14]. The dural substitute was placed in the aperture of a pressure chamber, mounted 10 cm below an access platform, which accommodated printed cylindrical access portals of 6 mm length and 14 mm or 22 mm diameter (Figures 3A, 3B). A 500 cc graduated cylinder was connected to the pressure chamber and the height of the water column accurately maintained pressures between 5-100 cm H_2_O to recapitulate CSF in human dura at normal and supranormal physiologic pressures. A durotomy was created with a standardized 10 mm cruciate incision in the membrane, and fluid leakage after dural repair collected at timed intervals in a reservoir. In the second group of experiments (below), a 3-D printed pressure chamber cassette with a curved dural surface was placed in the spinal canal of a 3-D printed lumbar spine model and an L4 laminotomy was performed such that the dura in the cassette was directly beneath the access portal at 30° offset (Figure 4).

The lumbar laminotomy model was secured within the simulator and connected to tubing from the graduated cylinder as above to simulate the variable pressures within the thecal sac and provide haptic feedback to the surgeon (Figure 4C). The access portals and the camera within the MIS box allowed a highly realistic simulation of endoscopic MIS durotomy repair including video visualization of the dura, restricted access corridors, and a curved dural surface with internal fluid under pressure.

Simulation of durotomy repair

The MIS simulator was used to test the repair and sealing efficacy for MIS repair of durotomy by two contemporary durotomy repair techniques (suture closure and inlay dural substitute graft) in comparison to a novel dural repair device (Patchclamp, Medtech, Seattle, WA). The Patchclamp device deploys a self-expanding synthetic or tissue graft on the inner surface of the dura and secures it in place using a bioabsorbable clasp on the outer surface of the dura (Figure 4). For both the pressure chamber and laminotomy models, a board-eligible neurosurgical fellow worked via a 14 mm port at either perpendicular or 30° offset in the MIS simulator, and utilized the endoscopic camera views to suture the dura or deploy the graft through the durotomy incision and secure it in place below the dura. Patchclamp's sealing efficacy was compared to two commonly utilized dural repair techniques: (a) suturing with 5-0 Neurolon (Ethicon; Rariton, NJ) supplemented by an onlay synthetic graft (DuraMatrix Onlay Plus, Stryker, Kalamazoo, MI) with fibrin sealant (Tisseel, Baxter/Synovis, St. Paul, MN); or (b) inlay graft (DuraMatrix Onlay Plus, Stryker, Kalamazoo, MI) manually placed below the dura under endoscopic vision and secured with fibrin sealant. Outcome measurements of the study included: (1) success of graft deployment through multiple tube diameters and port angles; (2) time (sec) required to complete the repair; (3) comparison of deployment times (sec) in the MIS simulator and spine model for the initial five versus last five deployments of both the Patchclamp and inlay grafts through multiple tube diameters and port angles.

Simulation of durotomy repair via pressure chamber and spine model endoscopic approaches

Although suture repair of the durotomy was successfully accomplished in an open, non-simulator pressure chamber model; repair via the MIS simulator was unsuccessful at all constrained simulator conditions due to limitations in portal size and working space at the durotomy, approach angle, and technical aspects of suture passage and knot tying. Figure 5 shows the median repair time for Patchclamp, suture, and inlay graft durotomy repairs in the open and MIS simulator models.

**Figure 5:**
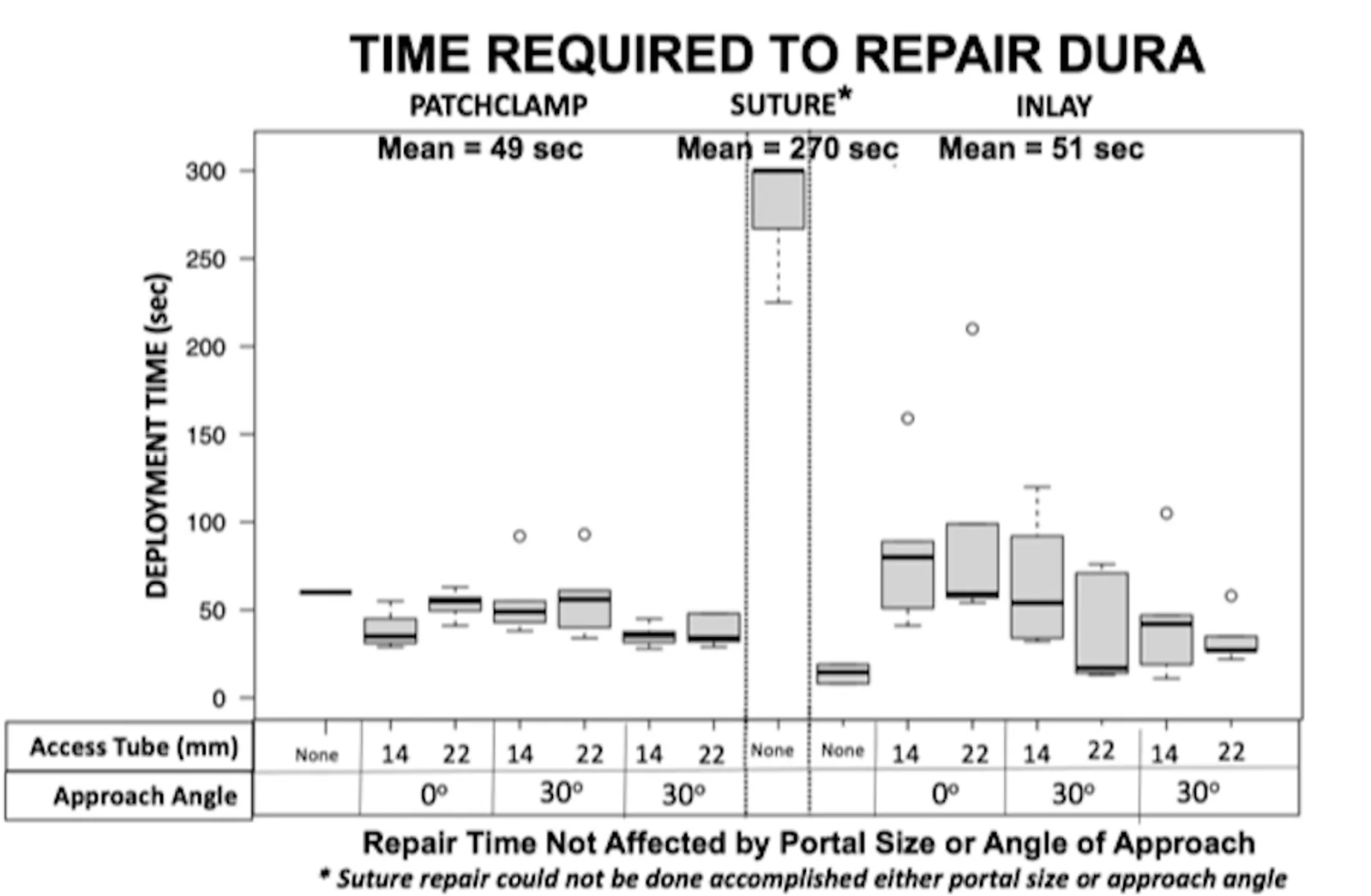
Time required to complete durotomy repair Time (seconds) required to complete the durotomy repair for the Patchclamp graft, suture and inlay graft for open simulator (No access or approach constraints) and minimally invasive surgery (MIS) simulator (combinations of limited access port and approach angle). Suture repair could not be accomplished in the MIS model. In the open model, time to repair was significantly less for Patchclamp and inlay compared to suture (p<0.01). Access portal size or approach angle did not affect the repair time for either technique.

Repair time with the Patchclamp was non-significantly less than inlay grafts (42.0 sec vs 52.2 sec; p=0.294). In the non-constrained (open surgery) mode, median repair time for inlay graft (41.5 sec; IQR=19-73sec) and Patchclamp (41 sec; IQR=34.0-55.0 sec) was significantly shorter than for suture repair (300 sec, IQR=248-308 sec; p<0.001). The operator was able to successfully deploy both the inlay graft and the Patchclamp graft through a 14 mm portal for both the 0° and 30° portals in the MIS simulator. Demonstration of the Patchclamp graft deployment, inlay graft deployment, and MIS suturing in the MIS simulator are shown in Video 1.

**Video 1:** MIS Simulator model of graft-clasp device deployment into the pressurized dural membrane via the hand-held applicator

## Discussion

MIS approaches across all surgical disciplines have become increasingly utilized due to reduced recovery times, lower costs, and improved satisfaction in select patients [1,2]. The substantial learning curve associated with MIS, coupled with limited low-cost opportunities for technical training, has impeded its adoption at large [3,4]. Thus, a lower-cost, easily reproducible model is desirable for the teaching of MIS techniques in multiple surgical specialties, especially in settings with limited resources. This may be particularly significant in global neurosurgery programs, where the overall benefit and savings of reducing CSF leaks would be substantial in comparison to the cost of the simulator. The MIS simulator described herein provides a low-cost platform for the teaching of endoscopic surgical techniques using 3-D printed models with realistic target tissue characteristics. 

Current surgical simulation models

Most models for surgical simulation involve cadavers, virtual/augmented reality, box trainers, or 3-D printed models [5-7]. While cadaveric courses provide haptic feedback and anatomically accurate soft tissue structures, utilization is limited by high cost and scarcity of facilities. Improvements in software have bolstered the use of virtual reality and augmented reality in surgical training. Although enhanced anatomical representations have been generated to simulate surgical approaches in a low-risk setting, virtual reality models may have an unexpected high cost associated with frequent software updates and maintenance of the simulators, as well as a lack of haptic feedback. Augmented reality simulators provide a unique opportunity by registering patient-specific data to the software to create precise 3-D virtual models to be used for surgical planning and refinement of motor skills, often including some aspect of haptic feedback [15]. However, widespread implementation of this technology has been hindered by its high cost and the tedious process of registering individual patient data in the software. Box trainers often provide the greatest breadth of haptic feedback, particularly in MIS endoscopic and laparoscopic cases. However, they often lack realistic features of human tissue and are limited to a small range of simulated procedures. A systematic review of the impact of surgical simulation on patient outcomes, including immediate and 30-day postoperative mortality and postoperative complication rates, found only one study utilizing a box trainer which had a significant impact in improving patient outcomes [16]. The primary deficiency of these models, however, is a lack of integration with MIS, particularly in the realm of cranial and spinal procedures.

Proof of concept of the MIS simulator

A realistic, low-cost, and easily reproduced simulator for MIS/endoscopic surgery is essential to promote technical proficiency for both trainees and established surgeons learning new MIS techniques and approaches. The MIS simulator described herein provides a realistic view of the operative windows present during endoscope-assisted or full endoscopic surgery. Additionally, as shown here, procedure-specific models, such as the 3-D printed spine model with pressurized thecal sac for MIS repair of durotomies, gives surgical trainees and those new to endoscopic techniques an opportunity to learn and practice procedures in a low-cost, low-risk setting. The preliminary trials with this model demonstrate improvement in durotomy repair time and efficacy compared to contemporary controls.

Prior physical models created for endoscopic spine surgical approaches have often lacked the ability to regulate pressure within the thecal sac or involved highly complex models which are often challenging and expensive to produce [8]. The MIS pressure chamber simulator and lumbar spine model with pressurized dura addresses this deficiency by accurately simulating the restricted windows of endoscopic spine surgery, intraoperative exposure with a curved dural surface, and providing the surgeon with haptic feedback from the pressurized dura within the spine model [17].

Cost involved in the optimization of surgical simulation

The most effective methods for surgical simulation involve realistic models of anatomy; real-time, haptic feedback for the user; a low cost to allow repetition; and a minimal need for technological updates. The most advanced models in current practice involve a combination of augmented reality and 3-D printed or xenograft models. Advanced models such as the LapSimâ and LapMentorâ (Surgical Science, Seattle, WA, USA) have shown great promise in simulating live tissue with real-time feedback to users [18]. When combined with annual software subscriptions and essential software updates, the costs of these models can range between $60,000 - $120,000, limiting their widespread integration. Mixed reality simulators such as the HoloLens (Microsoft, Seattle, WA) provide real-time, ergonomically favorable feedback to the surgeon while decreasing the need for expensive live models. However, the cost can still exceed $15,000 when all additional technologies are considered [19]. The current cost to produce the MIS simulator model is less than $300 when the 3-D printing material, servo infusion pump, and dural substitute are combined, making it broadly accessible to surgical training programs and trainees with limited resources.

Future directions

The scope of the MIS simulator extends beyond the realm of endoscopic spinal and cranial procedures. Many disciplines which implement MIS techniques such as orthopedics (arthroscopy), otolaryngology (sinus endoscopy), and general surgery/gynecology (laparoscopic abdomino-pelvic surgery) may benefit from the training experience with flexible ability to determine the degree of restricted access, limited operative windows, and procedure-specific surgical scenarios afforded by the MIS simulator. The simulator frame allows easy substitution of a simulated surgical target with unique features such the realistic pressurized dural membrane illustrated in the endoscopic spine simulation. Continued advancements in 3-D printing may allow for a range of models and tissues to be produced for procedure-specific training that recapitulate clinical experience. Improvements in tissue models to allow for both access and tissue haptic features will likely provide more realistic models for trainees [20]. In addition, the integration of artificial intelligence with this model may serve to provide immediate feedback and monitor technical progress of the user [14]. 

Limitations

This study is primarily limited by the early stages of its development. This novel simulator device has shown early promise and potential for modeling a wide range of surgical procedures; however, only a lumbar spine model with simulated dural tissue was used for validation in this study. Additionally, the MIS simulator and 3-D printing technologies includes a spine model with pressurized dural substitute, which lacks the surrounding soft tissues, bleeding, and real time physiologic responses to MIS. Finally, a single neurosurgical fellow performed the dural repairs; thus, there is limited data to show its efficacy across a wide range of users. Further study is needed in a larger cohort of participants with continued advancements to the model itself.

## Conclusions

The MIS simulator, with the 3-D printed spine model and pressurized dural membrane, provides a low-cost, easily reproducible model for teaching MIS techniques for durotomy repair. Early trials with the Patchclamp device have shown both its functional efficacy and the relative ease with which surgeons may assimilate its function in a simulation of the operative windows and restricted access present in MIS approaches. The cost, portability, reproducibility of experience, and flexibility of use suggest that this technology may be expanded beyond the scope of neurosurgery and spine surgery into other minimally invasive fields.
